# Supplementary material for: Population structure, genetic connectivity, and adaptation in the Olympia oyster (Ostrea lurida) along the west coast of North America
Source: Evol Appl. 2019 Feb 28;12(5):923–39. doi: 10.1111/eva.12766 (PMC6503834; doi:10.1111/eva.12766)
Supplement: Supplementary file 1 [file EVA-12-923-s001.pdf]

loci may be useful for characterizing the adaptive potential of a species or population to future environmental conditions (Eizaguirre and Baltazar-Soares, 2014). Inferences from both neutral and adaptive markers should be combined when making management recommendations (Funk et al., 2012).

The Olympia oyster (*Ostrea lurida*, Carpenter 1864) is a native estuarine bivalve found from Baja California to the central coast of Canada, patchily distributed over strong environmental gradients (Chan et al., 2017; Schoch et al., 2006). Oysters are ecosystem engineers in estuaries, providing structured habitat and removing suspended sediments (zu Ermgassen et al., 2013; Coen et al., 2011). Unlike other oysters where both males and females spawn gametes (e.g., *Crassostrea*), the females fertilize eggs with sperm from the water column and initially brood larvae in the mantle cavity. After release, the larvae have been reported to be planktonic from seven days to eight weeks before settling on a hard substrate (Baker, 1995). The impact of maternal brooding on population structure in Osterideae has not been examined.

Following devastating commercial exploitation in the 19th and early 20th centuries, recovery of Olympia oyster populations has been stifled by other anthropogenic threats (e.g., water quality issues, habitat loss, and possibly ocean acidification (Blake and Bradbury, 2012; Hettinger et al., 2013; Sanford et al., 2014)). The last 15 years has seen increased interest in the Olympia oyster, with restoration projects underway by both government and nongovernment agencies across its range (Pritchard et al., 2015). Current knowledge about the population genetic structure of *O. lurida* comes primarily from an unpublished 2011 dissertation, which sampled from San Francisco, CA to Vancouver Island, BC and found regional population structure using microsatellites (Stick, 2011). Two phylogeographic studies using two mitochondrial loci identified a phylogeographic break north of Willapa Bay, WA and established the southern boundary divide between *O. lurida* and its sister species *Ostrea conchaphila* (Polson et al., 2009; Raith et al., 2016). Future and ongoing management plans would benefit greatly from thorough analysis of the fine-scale genetic structure using modern genomic techniques and rangewide sampling (Camara and Vadopalas, 2009).

The objective of this study was to characterize the spatial population structure of the Olympia oyster across the majority of its range using both neutral and adaptive markers derived from genome-wide single nucleotide polymorphisms (SNPs). I specifically tested whether patterns of genetic variation suggest a smooth continuum of allele frequency shifts consistent with isolation-by-distance (IBD) (Malécot, 1968), regional blocks of genetic similarity that correspond to physical barriers (Hare and Avise, 1996), or the null model of no significant genetic differentiation (Grosberg and Cunningham, 2001). SNPs produced from high-throughput sequencing have led to the identification of previously undetected population structure in a number of marine

from Jon Puritz's lab (Puritz et al., 2014). Input files and formats for subsequent analysis of population structure were created using a combination of custom Python code, custom R code, and the *radiator* R package (Gosselin, 2017). Every step of the assembly, filtering process, and creation of input files can be reproduced through Jupyter notebooks.

Detection of loci under putative selection

Following recommendations to utilize multiple methods to detect loci under putative directional selection (Benestan et al., 2016; Rellstab et al., 2015), three approaches were used on the filtered SNP dataset: BayeScan v.2.1, *OutFLANK* v.0.2, and *pcadapt* v.4.0.2. For BayeScan and *OutFLANK*, individuals were grouped into populations by sampling site. GBS loci which had SNPs identified as outliers in at least two of the approaches were classified as putative adaptive GBS loci. From these GBS loci, any SNP that had been identified as an outlier by at least one approach was separated from the full SNP dataset to create an "outlier" SNP dataset. Subsequent analyses of population structure were conducted on three SNP datasets: all SNPs (combined), outlier SNPs, and neutral SNPs—which excluded any SNP found on a putative adaptive GBS locus.

BayeScan uses a Bayesian approach to apply linear regression to decompose F_{ST} coefficients into population- and locus-specific components and estimates the posterior probability of a locus showing deviation from Hardy–Weinberg proportions (Foll and Gaggiotti, 2008). BayeScan analysis was based on 1:100 prior odds, with 100,000 iterations, a burn-in length of 50,000, a false discovery rate (FDR) of 10%, and default parameters. Results were visualized in R. *OutFLANK* is an R package that identifies $\mathrm{F_{ST}}$ outliers by inferring a distribution of neutral F_{ST} using likelihood on a trimmed distribution of F_{ST} values. Because of its likelihood method, *OutFLANK* calculates F_{ST} without sample size correction when inferring the neutral distribution. Simulation studies have shown that this approach has lower false positive rates compared to other F_{ST} outlier methods (Whitlock and Lotterhos, 2015). *OutFLANK* was run using default parameters and a q-value threshold of 0.1, which can be considered a false discovery rate (FDR) of 10%. For the R package *pcadapt*, individuals are not sorted into predefined populations. Instead, *pcadapt* ascertains population structure using principal component analysis (PCA), then identifies markers under putative selection as those that are excessively correlated with population structure. When compared to BayeScan, *pcadapt* was shown to have greater power in the presence of admixed individuals and when population structure is continuous (Luu et al., 2017)—both scenarios which are likely in *O. lurida*. A scree plot representing the percentage of variance explained by each PC was used to choose the number of principal components (K) for *pcadapt*, and SNPs with a q-value threshold of 0.1 were categorized as outliers.

RESULTS

GBS and outlier detection

117 samples remained after removal of 14 samples with < 200,000 raw sequencing reads, 49 samples with < 15,000 clusters, and 65 samples missing data for over 55% of loci assembled across at least 75% of samples. One of the sampling sites for Willapa Bay, WA had a low number of individuals after filtering, so individuals from these two sites were combined into one population, for 19 total populations (4–9 individuals per population, mean = 6.2). 41,159 biallelic SNPs across 9,696 GBS loci were genotyped in greater than 75% of these individuals (2.8% of prefiltered loci assembled by *ipyrad*). Average read depth per individual per GBS locus ranged from 21 to 120 (mean = 32±14). Further filtering by HWE and MAF > 2.5% reduced the dataset to 13,424 SNPs across 6,187 GBS loci (the ”combined” dataset).

Three different methods were employed to identify putative SNPs under selection. The number of outliers detected by each program and the overlap between programs is illustrated in Figure D1. *OutFLANK*, as the most conservative of the programs used (Whitlock and Lotterhos, 2015), had the lowest number of outlier markers detected with 31 SNPs across 16 GBS loci. 29 SNPs found across 16 GBS loci were identified as outliers by all three programs. 129 GBS loci contained SNPs identified as outliers by at least two approaches, with 235 SNPs included in the outlier dataset for subsequent population structure analyses. The neutral dataset, with 13,073 SNPs across 6,057 GBS loci, excluded any SNP found on a GBS locus with an outlier SNP.

Summary statistics, population differentiation, and spatial structure

Summary statistics

Global F_{ST} for outliers (F_{ST} = 0.417) was almost four five times greater than for the combined and neutral SNPs (F_{ST}= 0.105 (combined), 0.097 (neutral)). The outlier dataset had the lowest H_o, but the highest H_e (Table 1). Average F_{IS} within populations for the combined dataset was 0.0424, with all populations having a significantly positive F_{IS} value except Ladysmith, BC, Tomales Bay, CA, and South San Francisco Bay, CA which had small, yet significantly negative F_{IS} values. Mugu Lagoon had the highest F_{IS} value (Table A1). Summary statistics for the six phylogeographic regions identified in the following section are show in Table B1. Summary statistics were quantitatively very similar for the combined and neutral datasets, so that only the results for the outlier and neutral datasets are reported for all subsequent analyses.

(2015); Riviere et al. (2013); Pauletto et al. (2017); Wang et al. (2018); Shiel et al. (2017); Pan et al. (2015); de Lorgeril et al. (2005). 21 additional outlier GBS loci had positive matches to InterPro signatures without any BLASTx hits or gene ontology annotation. Plotting minor allele frequency against latitude for outlier SNPs demonstrates that the majority of outliers show a clinal pattern, where one allele is fixed from either Coos Bay, OR or San Francisco Bay, CA to the north, and the other alelle increases in frequency towards the south (Figure D2).

DISCUSSION

Reduced-representation genomic methods, such as GBS, can greatly inform reintroduction efforts for threatened and exploited species by resolving fine-scaled population structure, providing estimates of genetic connectivity, and identifying informative markers for characterizing adaptive variation (Allendorf et al., 2010; Gagnaire et al., 2015). Using 13,424 GBS-derived SNPs, I characterized the rangewide population structure of the Olympia oyster from southern California to British Columbia and further identified 235 SNPs across 129 GBS loci potentially associated with local adaptation. Contrary to studies in some other marine species, neutral markers had greater power to detect fine-scale population structure compared to outliers. However, outlier loci did provide evidence for adaptive divergence among some populations with high inferred admixture, and are informative as candidate loci involved in local adaptation. This study highlights the importance of using both neutral and outlier markers for conservation and management applications.

Regional population structure and gene flow

Significant population structure was observed across the range of *O. lurida* in both the neutral and outlier markers, with sampling locations structured into six distinct regions. Notably, most of these regions fit well within previously described biogeographical provinces based on marine species distributions(Hall, 1964; Valentine, 1966; Fenberg et al., 2015). In addition to describing the rangewide population structure of *O. lurida*, the large geographic sampling of this study can facilitate the identification of oceanographic features along the eastern Pacific coast that may be important for structuring populations of marine species with similar life histories. Most of the inferred phylogeographic regions are bounded by areas of reduced gene flow, many of which align to oceanographic features that may be acting as barriers to dispersal. Below I discuss these phylogeographic regions and potential barriers in more detail, as well as provide some recommendations for management at local scales.

belong to a separate phylogeographic region all together, as this site was intermediate between *NWBC* and *Puget+BC* regions in the STRUCTURE, PCA, and TreeMix analyses. Genetic sampling from additional sites on the central coast of British Columbia and eastern coast of Vancouver Island could test this hypothesis.

The separation of these two regions from those to the south corroborates previous evidence from mitochondrial loci of a strong phylogeographic divide (Polson et al., 2009). Although Cape Flattery and Puget Sound itself have both been classified as biogeographic barriers due to a bifurcation in ocean currents (Valentine, 1966; Kelly and Palumbi, 2010), there are surprisingly few studies evaluating the genetic structure of species found both within Puget Sound and on the outer coast of Washington. Those that do focus on species with much longer dispersal times than *O. lurida* (Buonaccorsi et al., 2002; Cunningham et al., 2009; Iwamoto et al., 2015; Siegle et al., 2013; Jackson and O'Malley, 2017). To my knowledge, this is the first study in a marine mollusc to evaluate and identify significant population differentiation among Puget Sound populations and the outer coast. More studies are required to fully characterize the importance of this barrier across marine taxa.

Genetic differentiation within Puget Sound is relatively low at both neutral and outlier markers, with the exception of the northernmost site, Discovery Bay. The weak population structure within Puget Sound and the overall low genetic diversity in northern sites is likely due to recent genetic bottlenecks and range expansion after the last glacial maximum, which reached just north of Willipa Bay, WA (49°N latitude) until 12-13 kya (Dyke and Prest, 1987). Despite such low genetic differentiation, experimental assessments of local adaptation for populations within Puget Sound have detected heritable differences in fitness traits such as reproductive timing, growth rate, and gene expression in response to stress (Heare et al., 2017, 2018; Silliman et al., 2018). These results, coupled with experimental evidence for local adaptation to salinity among Northern California populations (Bible and Sanford, 2016), suggest that adaptive divergence in this species can occur in the face of high gene flow.

Anthropogenic influences on population structure

The evidence for reduced effective migration, low differentiation within most of the phylogeographic regions, and external estimates of effective dispersal (Carson, 2010), suggests that long distance dispersal is not a significant force in shaping population structure in this species. However, TreeMix inferred a few such migration events that cross aforementioned barriers to gene flow. To explain this evidence, I investigated the history of Olympia oyster exploitation and aquaculture through literature reviews, technical reports, grey literature, historical first-person accounts, and discussions with current restoration practitioners. The historical impact of human take and transportation on the Olympia oyster is substantial.

underlie the large number of individuals (128) removed during filtering. First, too many individuals may have been pooled per sequencing lane given the number of loci targeted, resulting in low sequencing depth for some individuals (Andrews et al., 2016). Second, these libraries were made and sequenced in-house as opposed to a dedicated commercial GBS facility. The protocol learning curve may be why a disproportionate number of individuals failed or had low sequencing depth in the first few prepared libraries. This filtering resulted in 4–9 individuals per population in the final dataset, which is sufficient for estimating F_{ST} when $> 1,000$ SNPs are used (Willing et al., 2012). While these small population sizes may limit the power to detect outlier loci (Foll and Gaggiotti, 2008), the probability of false positives is reduced by comparing across multiple outlier methods (Rellstab et al., 2015). Lastly, while methods like EEMS and PCA can characterize genetic differentiation, they cannot distinguish between the different demographic scenarios that may result in these patterns (Petkova et al., 2016).

CONCLUSIONS

This study provides the first comprehensive characterization of both neutral and adaptive population structure in the Olympia oyster, an ecologically important coastal species in North America. These results have direct implications for management policies and ongoing restoration efforts, and a future sustainable fishery. Putative adaptive loci identified here are excellent candidates for future research and may provide targets for genetic monitoring programs. Beyond these specific applications, this study contributes to the growing body of evidence for both population structure and adaptive differentiation in marine species. In particular, it is one of the first to utilize thousands of SNPs to characterize population structure from southern California to Vancouver Island. All analyses conducted for this study can be replicated using annotated Jupyter notebooks, allowing for clear dissemination of bioinformatics methods and future open-sourced research on the population structure of *O. lurida*.

DATA ARCHIVING STATEMENT

Genomic data (all filtered markers, putative neutral markers, and putative outliers) and sample metadata are available on Dryad (https://doi.org/10.5061/dryad.114j8m1). Raw demultiplexed DNA sequences for all sequenced individuals with $> 200,000$ raw sequencing reads are available on NCBI SRA (Project Accession Number: PRJNA511386). Reproducible Jupyter notebooks are available at https://github.com/ksil91/Ostrea_PopStructure.

the ecological services provided by and impacts of native and cultured bivalves in shellfish-dominated ecosystems. In Shumway, S. E., editor, *Shellfish Aquaculture and the Environment*, chapter 9, pages 239–295. John Wiley & Sons, Inc.

Cowen, R. K., Lwiza, K. M., Sponaugle, S., Paris, C. B., and Olson, D. B. (2000). Connectivity of marine populations: Open or closed? *Science*, 287:857–859.

Cunningham, K. M., Canino, M. F., Spies, I. B., and Hauser, L. (2009). Genetic isolation by distance and localized fjord population structure in Pacific cod (*Gadus macrocephalus*): Limited effective dispersal in the northeastern Pacific Ocean. *Can. J. Fish. Aquat. Sci.*, 66(1):153–166.

Danecek, P., Auton, A., Abecasis, G., Albers, C. A., Banks, E., DePristo, M. A., Handsaker, R. E., Lunter, G., Marth, G. T., Sherry, S. T., McVean, G., Durbin, R., and 1000 Genomes Project Analysis Group (2011). The variant call format and VCFtools. *Bioinformatics*, 27(15):2156–2158.

David, A. A. (2018). Reconsidering panmixia: The erosion of phylogeographic barriers due to anthropogenic transport and the incorporation of biophysical models as a solution. *Frontiers in Marine Science*, 5:280.

Dawson, M. N. (2001). Phylogeography in coastal marine animals: A solution from California? *Journal of Biogeography*, 28(6):723–736.

de Lorgeril, J., Saulnier, D., Janech, M. G., Gueguen, Y., and Bachère, E. (2005). Identification of genes that are differentially expressed in hemocytes of the pacific blue shrimp (*Litopenaeus stylirostris*) surviving an infection with vibrio penaeicida. *Physiol. Genomics*, 21(2):174–183.

De Wit, P. and Palumbi, S. R. (2013). Transcriptome-wide polymorphisms of red abalone (*Haliotis rufescens*) reveal patterns of gene flow and local adaptation. *Mol. Ecol.*, 22(11):2884–2897.

Drinan, D. P., Gruenthal, K. M., Canino, M. F., Lowry, D., Fisher, M. C., and Hauser, L. (2018). Population assignment and local adaptation along an isolation-by-distance gradient in Pacific cod (*Gadus macrocephalus*). *Evol. Appl.*, 103:17302.

Dyke, A. S. and Prest, V. K. (1987). Late Wisconsinan and Holocene history of the Laurentide ice sheet. *Gogr. Phys. Quat.*, 41(2):237.

Earl, Dent A. and vonHoldt, Bridgett M (2012). STRUCTURE HARVESTER: a website and program for visualizing STRUCTURE output and implementing the evanno method. *Conserv. Genet. Resour.*, 4(2):359–361.

Eaton, D. A. R. (2014). PyRAD: assembly of de novo RADseq loci for phylogenetic analyses. *Bioinformatics*, 30(13):1844–1849.

structure in the Salish Sea. *ICES J. Mar. Sci.*, 72(9):2720–2731.

Jackson, T. M. and O'Malley, K. G. (2017). Comparing genetic connectivity among dungeness crab (*Cancer magister*) inhabiting puget sound and coastal washington. *Mar. Biol.*, 164(6):123.

Jombart, T. and Ahmed, I. (2011). adegenet 1.3-1: new tools for the analysis of genome-wide SNP data. *Bioinformatics*, 27(21):3070–3071.

Kanwal, S., Khan, F. Z., Lonie, A., and Sinnott, R. O. (2017). Investigating reproducibility and tracking provenance - a genomic workflow case study. *BMC Bioinformatics*, 18(1):337.

Kelly, R. P. and Palumbi, S. R. (2010). Genetic structure among 50 species of the northeastern Pacific rocky intertidal community. *PLoS One*, 5(1):e8594.

Kluyver, T., Ragan-Kelley, B., Pérez, F., Granger, B., Bussonnier, M., Frederic, J., Kelley, K., Hamrick, J., Grout, J., Corlay, S., Ivanov, P., Avila, D., and Jupyter Development Team (2016). Jupyter notebooks — a publishing framework for reproducible computational workflows. In Loizides, F. and Schmidt, B., editors, *Positioning and Power in Academic Publishing: Players, Agents and Agendas*, pages 87–90. IOS Press.

Kopelman, N. M., Mayzel, J., Jakobsson, M., Rosenberg, N. A., and Mayrose, I. (2015). CLUMPAK: A program for identifying clustering modes and packaging population structure inferences across K. *Mol. Ecol. Resour.*, 15(5):1179–1191.

Lallias, D., Taris, N., Boudry, P., Bonhomme, F., and Lapègue, S. (2010). Variance in the reproductive success of flat oyster ostrea edulis l. assessed by parentage analyses in natural and experimental conditions. *Genet. Res.*, 92(3):175–187.

Larson, W. A., Seeb, L. W., Everett, M. V., Waples, R. K., Templin, W. D., and Seeb, J. E. (2014). Genotyping by sequencing resolves shallow population structure to inform conservation of Chinook salmon (*Oncorhynchus tshawytscha*). *Evol. Appl.*, 7(3):355–369.

Lenormand, T. (2002). Gene flow and the limits to natural selection. *Trends Ecol. Evol.*, 17(4):183–189.

Li, B., Song, K., Meng, J., Li, L., and Zhang, G. (2017). Integrated application of transcriptomics and metabolomics provides insights into glycogen content regulation in the Pacific oyster *Crassostrea gigas*. *BMC Genomics*, 18(1):713.

Lowry, D. B., Hoban, S., Kelley, J. L., Lotterhos, K. E., Reed, L. K., Antolin, M. F., and Storfer, A. (2017). Breaking RAD: an evaluation of the utility of restriction site-associated DNA sequencing for genome scans of adaptation. *Mol. Ecol. Resour.*, 17(2):142–152.

Luisetti, T., Turner, R. K., Jickells, T., Andrews, J., Elliott, M., Schaafsma, M., Beaumont, N., Malcolm, S.,

Schoch, G. C., Menge, B. A., Allison, G., Kavanaugh, M., Thompson, S. A., and A. Wood, S. (2006). Fifteen degrees of separation: Latitudinal gradients of rocky intertidal biota along the California Current. *Limnol. Oceanogr.*, 51(6):2564–2585.

Shiel, B. P., Hall, N. E., Cooke, I. R., Robinson, N. A., and Strugnell, J. M. (2017). Epipodial tentacle gene expression and predetermined resilience to summer mortality in the commercially important greenlip abalone, haliotis laevigata. *Mar. Biotechnol.*, 19(2):191–205.

Siegle, M. R., Taylor, E. B., Miller, K. M., Withler, R. E., and Yamanaka, K. L. (2013). Subtle population genetic structure in yelloweye rockfish (*Sebastes ruberrimus*) is consistent with a major oceanographic division in British Columbia, Canada. *PLoS One*, 8(8):e71083.

Silliman, K. E., Bowyer, T. K., and Roberts, S. B. (2018). Consistent differences in fitness traits across multiple generations of Olympia oysters. *Sci. Rep.*, 8(1):6080.

Sokal, R. R. (1979). Testing statistical significance of geographic variation patterns. *Syst. Zool.*, 28(2):227–232.

Stapley, J., Reger, J., Feulner, P. G. D., Smadja, C., Galindo, J., Ekblom, R., Bennison, C., Ball, A. D., Beckerman, A. P., and Slate, J. (2010). Adaptation genomics: The next generation. *Trends Ecol. Evol.*, 25(12):705–712.

Stick, D. A. (2011). *Identification of optimal broodstock for Pacific Northwest oysters*. PhD thesis, Oregon State University.

Szent-Györgyi, A. G., Kalabokis, V. N., and Perreault-Micale, C. L. (1999). Regulation by molluscan myosins. In Imai, S., Ohtsuki, I., and Endo, M., editors, *Muscle Physiology and Biochemistry*, pages 55–62. Springer US, Boston, MA.

Tepolt, C. K. and Palumbi, S. R. (2015). Transcriptome sequencing reveals both neutral and adaptive genome dynamics in a marine invader. *Mol. Ecol.*, 24(16):4145–4158.

Townsend, C. H. (1895). The transplanting of eastern oysters to Willapa Bay, Washington, with notes on the native oyster industry. Technical Report 334, U.S. Bureau of Fisheries.

Valentine, J. W. (1966). Numerical analysis of marine molluscan ranges on extratropical northeastern Pacific shelf. *Limnol. Oceanogr.*, 11:198–211.

Van Wyngaarden, M., Snelgrove, P. V. R., DiBacco, C., Hamilton, L. C., Rodríguez-Ezpeleta, N., Jeffery, N. W., Stanley, R. R. E., and Bradbury, I. R. (2016). Identifying patterns of dispersal, connectivity, and selection in the sea scallop, *Placopecten magellanicus,* using RAD-seq derived SNPs. *Evol. Appl.*, (August).

Locus ID	Gene description	Top GO IDs	Top hit species
locus_5648	DNA N6-methyl adenine demethylase	F:dioxygenase activity activity	*C. gigas*
locus_6412	glucose dehydrogenase [FAD, quinone]	None	*C. gigas*
locus_7299	transcriptional regulator ERG	None	*C. gigas*
locus_10670	Fez family zinc finger protein 1	F:nucleic acid binding	*C. gigas*
locus_44811	sodium-dependent phosphate transport protein 2B	F:sodium-dependent phosphate transmembrane transporter activity	*C. gigas*
locus_50945	glyoxalase 3-like	None	*C. virginica*
locus_57217	uncharacterized protein LOC111115623	None	*C. virginica*
locus_98257	uncharacterized protein LOC111133343	None	*C. virginica*
locus_121489	E3 ubiquitin-protein ligase TRIM9	F:zinc ion binding	*C. virginica*
locus_123004	Transposon Ty3-G Gag-Pol polyprotein	None	*Mizuhopecten yessoensis*
locus_170867	carnitine O-palmitoyltransferase 2, mitochondrial	F:calcium ion binding, F: transferase activity	*C. gigas*
locus_196263	myosin-XVIIIa	F:actin filament binding	*C. gigas*
locus_251628	myosin heavy chain, striated muscle	F:microtubule motor activity	*C. gigas*
locus_252560	helicase domino-like	None	*C. virginica*
locus_276278	heavy metal-binding protein HIP	None	*C. gigas*
locus_277490	NADH dehydrogenase subunit 5, mitochondrion	C:mitochondrion	*O. lurida*
locus_339584	serine/threonine-protein kinase B-raf	F:metal ion binding, F:kinase activity, P:intracellular signal transduction	*C. virginica*
locus_339916	vesicular glutamate transporter 2.1	P:transmembrane transport	*C. gigas*

Table 2. BLASTx and gene ontology (GO) annotation results for outlier loci. Only the 18 loci with positive BLAST hits are shown. F: molecular function, C: cellular component, P: biological process.

.

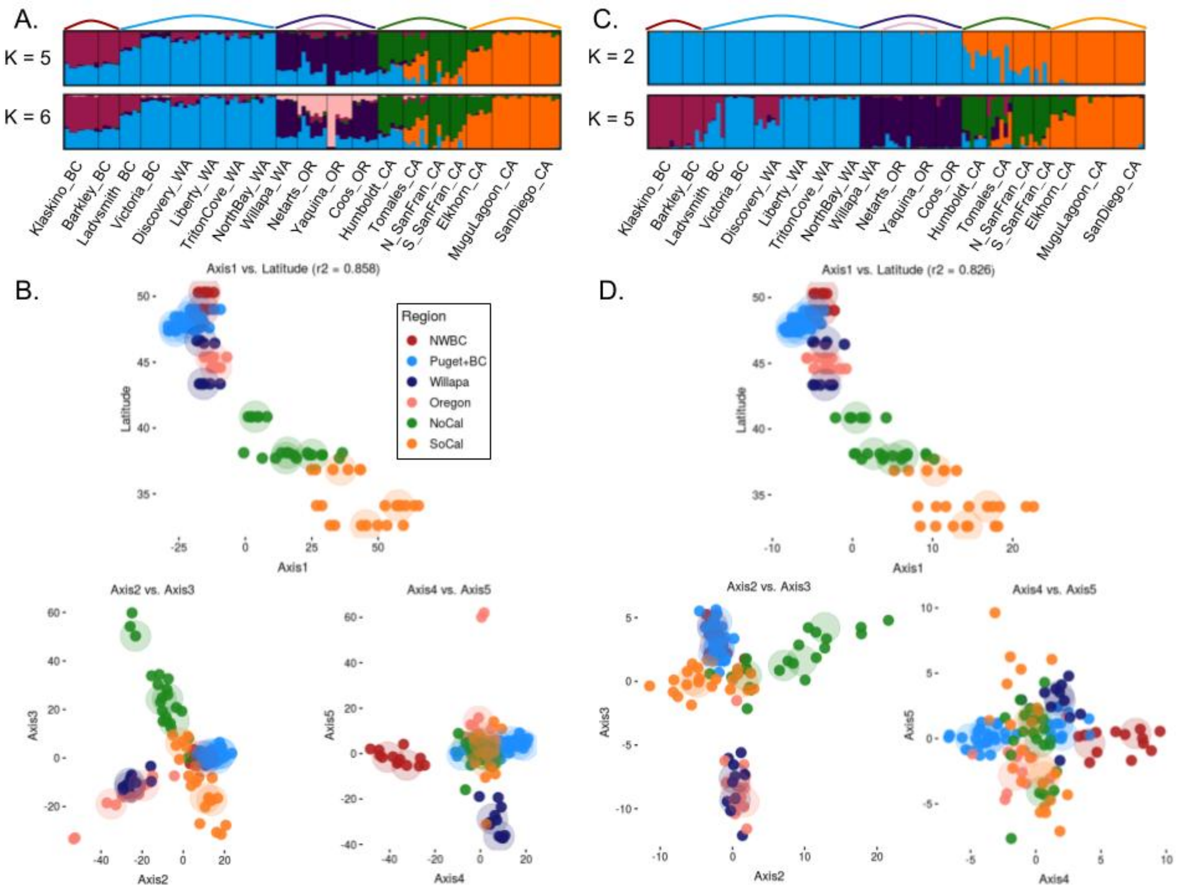

Figure 2

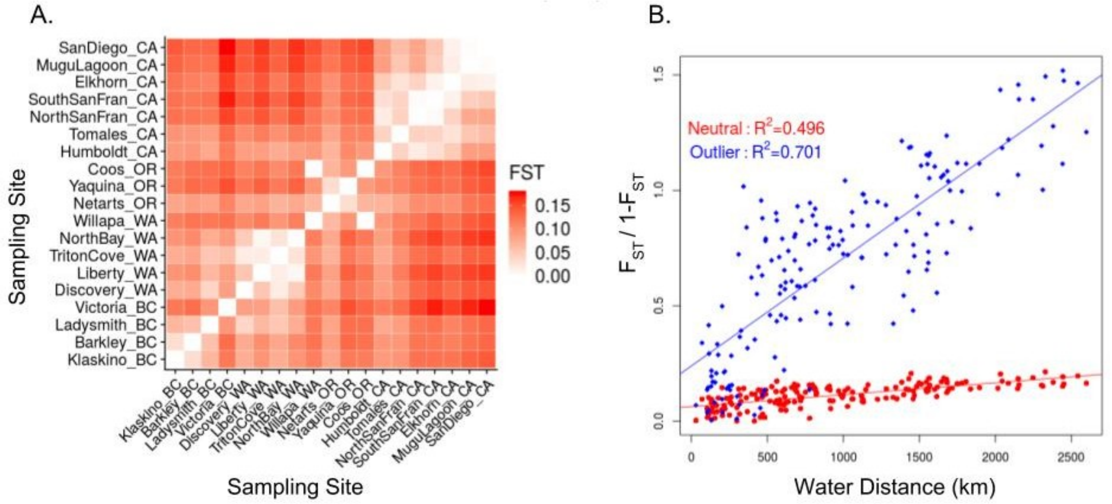

Figure 3

APPENDICES

Appendix A: Sampling locations and population-specific summary statistics

Sampling Site	Latitude	Longitude	# of individuals used in analysis	H_e	F_{IS} (C.I.)
Klaskino Inlet, BC	50.29867	-127.72363	8	0.1903	0.0686 (0.0594 - 0.0786)
Barkley Sound, BC	49.01585	-125.31417	5	0.1865	0.0.0664 (0.0540 - 0.0795)
Ladysmith Harbour, BC	49.01138	-123.8357	5	0.1897	−0.0554 (−0.0700 - −0.0396)
Victoria Gorge, BC	48.43567	-123.37791	7	0.1717	0.0515 (0.0405 - 0.0631)
Discovery Bay, Puget Sound, WA	47.9978	-122.8824	7	0.1810	0.0593 (0.0483 - 0.0699)
Liberty Bay, Puget Sound, WA	47.7375	-122.6507	6	0.1768	0.0309 (0.0194 - 0.0426)
Triton Cove, Puget Sound, WA	47.6131	-122.982	6	0.1820	0.0336 (0.0219 - 0.0462)
North Bay, Puget Sound, WA	47.3925	-122.8138	6	0.1756	0.0524 (0.0404 - 0.0634)
Willapa Bay, WA (North & South)	46.62477<br>46.4400	-123.98879<br>-124.004	3<br>2	0.1798	0.0556 (0.0420 - 0.0685)
Netarts Bay, OR	45.39116	-123.95590	7	0.1968	0.0584 (0.0470 - 0.0698)
Yaquina Bay, OR	44.57954	-123.99577	6	0.1876	0.0143 (0.0018 - 0.0274)
Coos Bay, OR	43.35599	-124.19316	6	0.1809	0.0531 (0.0411 - 0.0654)
Humboldt Bay, CA	40.85580	-124.09746	6	0.2146	0.0327 (0.0209 - 0.0451)
Tomales Bay, CA	38.11755	-122.87450	6	0.2270	−0.0023 (−0.0133 - 0.0077)
Point Orient, San Francisco Bay, CA	37.95507	-122.42180	5	0.2209	0.0560 (0.0450 - 0.0668)
Candlestick Park, San Francisco Bay, CA	37.70867	-122.37761	4	0.2234	−0.0974 (−0.1181 - −0.0745)
Elkhorn Slough, CA	36.83982	-121.74278	6	0.2477	0.0859 (0.0745 - 0.0978)
Mugu Lagoon, CA	34.10191	-119.10434	9	0.2535	0.1327 (0.1239 - 0.1411)
San Diego Bay, CA	32.60250	-117.11889	7	0.2500	0.0948 (0.0851 - 0.1049)

Table A.1. GPS coordinates of sampling sites and population-specific summary statistics averaged across markers using the combined dataset of 13,424 SNPs. H_e, expected heterozygosity; F_{IS}, inbreeding coefficient within the population, mean and 25%-75% confidence intervals (Nei and Chesser, 1983);

Appendix B: Summary statistics for phylogeographic regions

Region	H_o	H_e	F_{IS}	F_{ST}
NWBC	0.177	0.193	0.0821 (0.0738 - 0.0897)	0.016
Puget+BC	0.174	0.189	0.0814 (0.0758 - 0.0862)	0.046
Willapa	0.171	0.182	0.0583 (0.0495 - 0.0666)	0.001
Oregon	0.185	0.196	0.0556 (0.0474 - 0.0645)	0.016
NoCal	0.215	0.227	0.0536 (0.0472 - 0.0592)	0.022
SoCal	0.224	0.253	0.115 (0.1097 - 0.1209)	0.007

Table B.1. Overall summary statistics for each phylogeographic region using the neutral dataset of 13,073 SNPs. H_o, observed heterozygosity averaged across loci; H_e, expected heterozygosity averaged across loci; F_{IS} & F_{ST}, Wright's F-statistics averaged across loci (Nei and Chesser, 1983). Note that F_{ST} may be skewed by variation in sampling strategy across regions.

Appendix C: Pairwise FST values for outlier and neutral datasets

	Klaskino_BC	Barkley_BC	Ladysmith_BC	Victoria_BC	Discovery_WA	Liberty_WA	TritonCove_WA	NorthBay_WA	Willapa_WA	Netarts_OR	Yaquina_OR	Coos_OR	Humboldt_CA	Tomales_CA	NorthSanFran_CA	SouthSanFran_CA	Elkhorn_CA	MuguLagoon_CA
Klaskino_BC	0																	
Barkley_BC	0.0306	0																
Ladysmith_BC	0.0663	0.0564	0															
Victoria_BC	0.1193	0.1270	0.0931	0														
Discovery_WA	0.0742	0.0768	0.0381	0.0873	0													
Liberty_WA	0.0965	0.1021	0.0650	0.0864	0.0526	0												
TritonCove_WA	0.0751	0.0800	0.0516	0.0575	0.0176	0.0166	0											
NorthBay_WA	0.0965	0.1050	0.0750	0.0904	0.0476	0.0075	0.0246	0										
Willapa_WA	0.1136	0.1228	0.1197	0.1247	0.1056	0.1184	0.0946	0.1141	0									
Netarts_OR	0.0825	0.0888	0.0824	0.1097	0.0804	0.0854	0.0703	0.0777	0.0642	0								
Yaquina_OR	0.1190	0.1304	0.1276	0.1480	0.1220	0.1379	0.1184	0.1359	0.0948	0.0310	0							
Coos_OR	0.1159	0.1245	0.1265	0.1338	0.1120	0.1217	0.1048	0.1189	0.00139	0.0687	0.0918	0						
Humboldt_CA	0.0915	0.0936	0.0789	0.1172	0.0884	0.0877	0.0767	0.0870	0.0927	0.0684	0.1045	0.0927	0					
Tomales_CA	0.0970	0.0940	0.0886	0.1282	0.1033	0.1165	0.0998	0.1171	0.1122	0.0821	0.1116	0.1117	0.0397	0				
NorthSanFran_CA	0.1248	0.1222	0.1198	0.1555	0.1309	0.1452	0.1252	0.1458	0.1207	0.1023	0.1244	0.1280	0.0176	0.0418	0			
SouthSanFran_CA	0.1256	0.1204	0.1258	0.1717	0.1424	0.1566	0.1347	0.1574	0.1364	0.1025	0.1318	0.1364	0.0295	0.0399	0.0039	0		
Elkhorn_CA	0.1228	0.1135	0.1108	0.1578	0.1292	0.1431	0.1182	0.1417	0.1231	0.1004	0.1247	0.1284	0.0470	0.0261	0.0398	0.0135	0	
MuguLagoon_CA	0.1351	0.1250	0.1241	0.1698	0.1414	0.1559	0.1366	0.1588	0.1402	0.1212	0.1386	0.1448	0.0880	0.0523	0.0779	0.0453	0.0164	0
SanDiego_CA	0.1417	0.1322	0.1320	0.1764	0.1445	0.1619	0.1392	0.1641	0.1481	0.1270	0.1467	0.1534	0.0892	0.0604	0.0811	0.0520	0.0132	0.0016

Table C.1. Pairwise F_{ST} values for all pairs of populations, using the neutral dataset of 13,073 SNPs.

	Klaskino_BC	Barkley_BC	Ladysmith_BC	Victoria_BC	Discovery_WA	Liberty_WA	TritonCove_WA	NorthBay_WA	Willapa_WA	Netarts_OR	Yaquina_OR	Coos_OR	Humboldt_CA	Tomales_CA	NorthSanFran_CA	SouthSanFran_CA	Elkhorn_CA	MuguLagoon_CA
Barkley_BC	0.0660	0																
Ladysmith_BC	0.1848	0.0990	0															
Victoria_BC	0.4565	0.3553	0.3192	0														
Discovery_WA	0.2161	0.1151	0.0804	0.2707	0													
Liberty_WA	0.3705	0.2820	0.1894	0.1837	0.1306	0												
TritonCove_WA	0.3817	0.2981	0.1886	0.1616	0.1336	0.0398	0											
NorthBay_WA	0.3863	0.2874	0.2008	0.1777	0.1090	0.0109	0.0524	0										
Willapa_WA	0.4510	0.4372	0.4018	0.5178	0.3687	0.4915	0.4768	0.4871	0									
Netarts_OR	0.4119	0.3803	0.3214	0.3964	0.3247	0.3686	0.3832	0.3678	0.1496	0								
Yaquina_OR	0.4554	0.4401	0.3936	0.4739	0.4003	0.4548	0.4589	0.4515	0.2160	0.0453	0							
Coos_OR	0.4497	0.4396	0.3987	0.5085	0.3737	0.4934	0.4808	0.4839	0.0090	0.1328	0.2212	0						
Humboldt_CA	0.4659	0.4289	0.3807	0.5065	0.4316	0.4681	0.4676	0.4614	0.4397	0.3945	0.4271	0.4461	0					
Tomales_CA	0.3696	0.3283	0.3357	0.4652	0.3636	0.4571	0.4369	0.4443	0.3772	0.3299	0.3298	0.3935	0.2466	0				
NorthSanFran_CA	0.5057	0.4690	0.4456	0.5594	0.4951	0.5467	0.5366	0.5298	0.4958	0.4557	0.4642	0.4979	0.0475	0.2435	0			
SouthSanFran_CA	0.4822	0.4257	0.4303	0.5647	0.4762	0.5484	0.5446	0.5416	0.4800	0.4352	0.4515	0.4941	0.0830	0.1520	0.0637	0		
Elkhorn_CA	0.4782	0.4275	0.4407	0.5410	0.4716	0.5444	0.5343	0.5341	0.4457	0.4341	0.4413	0.4716	0.3276	0.1446	0.2733	0.1697	0	
MuguLagoon_CA	0.5749	0.5378	0.5490	0.6299	0.5760	0.6278	0.6190	0.6228	0.5608	0.5531	0.5555	0.5825	0.5130	0.3423	0.4705	0.3567	0.1320	0
SanDiego_CA	0.5744	0.5313	0.5471	0.6320	0.5758	0.6278	0.6194	0.6308	0.5700	0.5466	0.5507	0.5822	0.5059	0.3278	0.4753	0.3366	0.1271	0.0172

Table C.2. Pairwise F_{ST} values for all pairs of populations, using the outlier dataset of 235 SNPs.

Appendix D: Additional results of outlier analyses

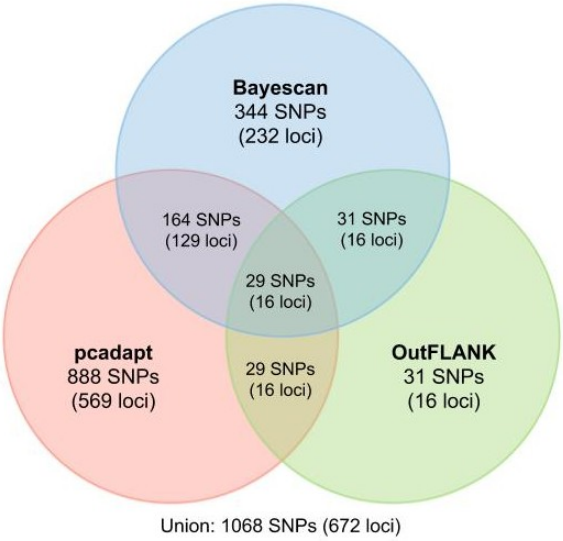

Figure D.1. Venn diagram with number of SNPs and Genotype-by-Sequencing loci identified as outliers by three methods: *pcadapt*, *OutFLANK*, and BayeScan

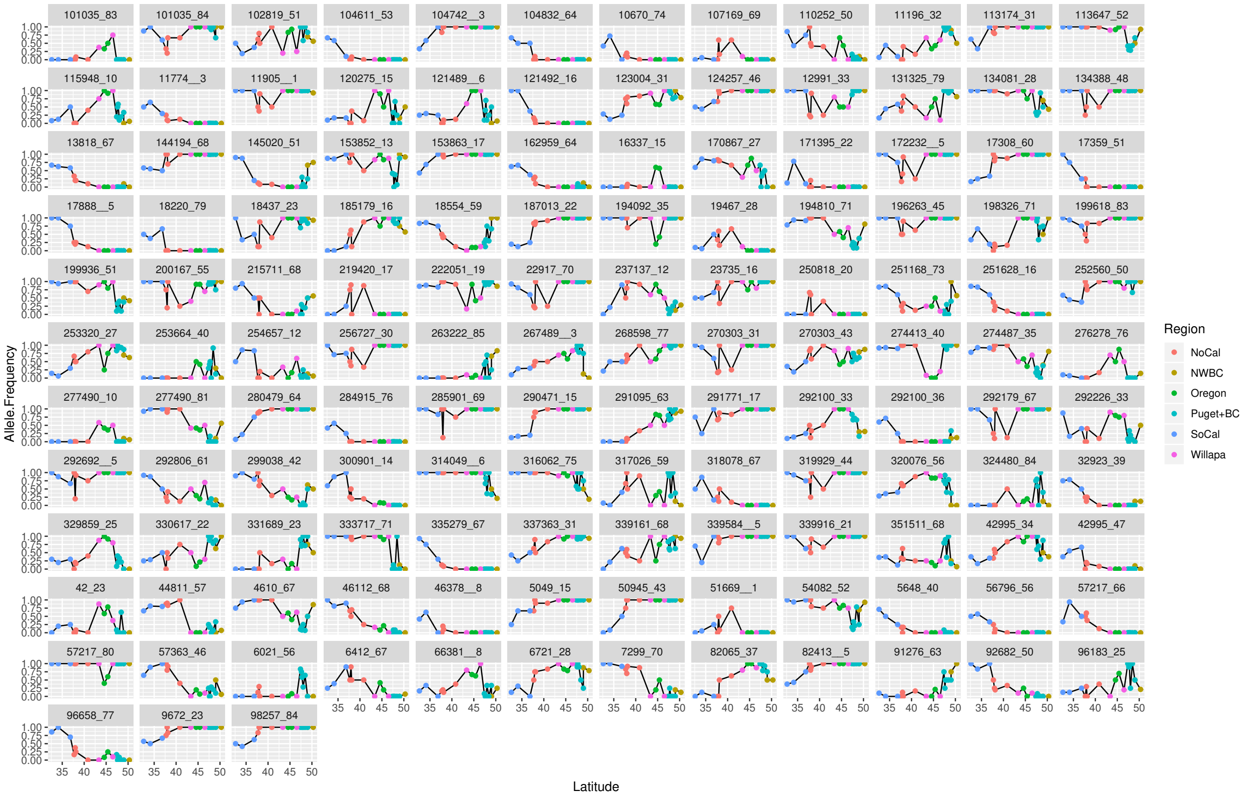

Figure D.2. Outlier loci predominantly show clinal patterns in allele frequency. Allele frequency in 129 individual outlier loci plotted against latitude for 19 populations of *O. lurida*. One SNP is represented for each locus, except in the case where two outlier SNPs from the same locus showed different spatial patterns (e.g., locus_277490). Populations are colored by inferred phylogeographic regions.
